# School gardening increases knowledge of primary school children on edible plants and preference for vegetables

**DOI:** 10.1002/fsn3.758

**Published:** 2018-09-07

**Authors:** Jasper R. F. W. Leuven, Annerie H. M. Rutenfrans, Alexander G. Dolfing, Rob S. E. W. Leuven

**Affiliations:** ^1^ Faculty of Geosciences Utrecht University Utrecht The Netherlands; ^2^ Beleef & Weet Consultancy for Education on Sustainability Nijmegen The Netherlands; ^3^ Natuur & Milieu Society for Nature and the Environment Utrecht The Netherlands; ^4^ Department of Environmental Science Radboud University Nijmegen The Netherlands

**Keywords:** children, knowledge, preference, primary school, school gardening, vegetable

## Abstract

At least 10% of children worldwide are diagnosed with overweight. Part of this problem is attributed to low vegetable intake, for which preference at a younger age is an indicator. Few studies examined long‐term effects of school garden interventions on the knowledge about and preference for vegetables. Therefore, in this study, an intervention period of 7 months (17 lessons) was organized for primary school students (*n* = 150) of age 10–12 years in the Municipality of Nijmegen (the Netherlands). Surveys were conducted before and after the intervention period to test the ability of students to identify vegetables, to measure their self‐reported preference for vegetables, and to analyze students’ attitudes toward statements about gardening, cooking, and outdoor activity. The long‐term effects were measured by repeating the survey 1 year after the intervention (*n* = 52). Results were compared with a control group of students (*n* = 65) with similar background and tested for significance with *α* = 0.05. School gardening significantly increases the knowledge of primary schoolchildren on 10 vegetables as well as their ability to self‐report preference for the vegetables. The short‐term (*n* = 106) and long‐term (*n* = 52) preference for vegetables increased (*p* < 0.05) in comparison with the control group. The latter did not show a significant learning effect (*p* > 0.05). This implies that the exposure to vegetables generated by school gardening programs may increase willingness to taste and daily intake of vegetables on the long term. Students’ attitudes toward gardening, cooking, and outdoor activity were unaffected by the intervention.

## 1 INTRODUCTION

The occurrence of childhood obesity is high and still increasing worldwide (Lobstein, Baur, & Uauy, 2004; Schroeder, Travers, & Smaldone, 2016). At least ten percent of the children worldwide are diagnosed with overweight, which results in increased risks for developing chronic diseases such as diabetes and sleep apnea (Ebbeling, Pawlak, & Ludwig, 2002; Lobstein et al., 2004; Schroeder et al., 2016). These diseases cause an increasing pressure on the health services, which enhances the governmental awareness of the obesity problem and the political willingness for policymaking to improve the food habits among children.

Part of the obesity problem is attributed to a low intake of fruits and vegetables and excessive inactivity among children (Ebbeling et al., 2002). Increasing the fruit and vegetable intake could largely reduce many noncommunicable diseases (Liu, 2003; Lock, Pomerleau, Causer, Altmann, & McKee, 2005) and childhood obesity (Davis, Ventura, Cook, Gyllenhammer, & Gatto, 2011; Ebbeling et al., 2002). School‐based interventions are suggested to improve the consumption of fruits and vegetables and promote physical activity (Ebbeling et al., 2002).

School gardening programs are thought to contribute to a healthy lifestyle, because they may increase preferential selection and willingness to taste fruits and vegetables (Birch, McPhee, Shoba, Pirok, & Steinberg, 1987), as well as to promote outdoor activities. It is likely that vegetable taste preferences of children extend to future vegetable consumption (Birch, 1979; Blanchette & Brug, 2005; Domel et al., 1996). Previous research showed that gardening at home (Faber, Phungula, Venter, Dhansay, & Benadé, 2002; Schreinemachers et al., 2015), in the community (Carney et al., 2012), or at school (Morgan et al., 2010; Morris & Zidenberg‐Cherr, 2002; Ratcliffe, Merrigan, Rogers, & Goldberg, 2011; Wright & Rowell, 2010) may increase the preference and potentially the intake of vegetables.

Increasing evidence from short‐term qualitative and quantitative data shows the positive effects of participation in “healthy school” programmes (Keyte, Harris, Margetts, Robinson, & Baird, 2012) and school gardens (Alexander, North, & Hendren, 1995; Bowker & Tearle, 2007; Cutter‐Mackenzie, 2009; Davis et al., 2011; Heim, Stang, & Ireland, 2009; Hermann et al., 2006; Morgan et al., 2010; Morris & Zidenberg‐Cherr, 2002; Ratcliffe et al., 2011; Wright & Rowell, 2010) on health of 5‐ to 14‐year‐old students (Supporting Information Table S1). However, the long‐term effect is unknown, because long‐term follow‐up is rarely undertaken (Appleton et al., 2016). In total, 13 studies reported school gardening interventions with sample size varying between *n* = 34 (Davis et al., 2011) and *n* = 234 (Wright & Rowell, 2010) and intervention duration varying between 3 weeks (Wright & Rowell, 2010) and 35 weeks (Nury, Sarti, Dijkstra, Seidell, & Dedding, 2017) (Supporting Information Table S1). Eleven studies were performed in Northern America, one in Australia, and one in Europe.

It is still challenging to prove that gardening in schools indeed contributes to a healthy lifestyle or, for example, a reduction in obesity cases, because only two studies continued monitoring after the intervention period (Morgan et al., 2010; Morris & Zidenberg‐Cherr, 2002), with a maximum duration of 6 months (Morris & Zidenberg‐Cherr, 2002). In the latter case, 81 students (age 9–10 years) participated in the intervention of which 63 completed the survey. Several reviews conclude that there may indeed be multiple positive effects from integrating gardening into a school setting (Appleton et al., 2016; Blair, 2009; Canaris, 1995). However, additional studies on the long‐term effect of gardening on vegetable preference, intake, and outdoor activity are requested (Appleton et al., 2016). More studies are required to extend the hypotheses from short‐term projects to the long‐term effect.

The aim here was to determine the effect of school gardening on (a) the ability to identify vegetables and the preference for vegetables of primary schoolchildren, and (b) their attitude toward healthy food, nature, gardening, and outdoor activities. Therefore, we implemented a school garden intervention of 17 lessons for a period of 7 months at three different schools and compared the results with a control group. The short‐ and long‐term effects were monitored by a survey immediately after the intervention and by a 1‐year follow‐up.

We hypothesize that involvement of children in school gardening (a) increases their capability to identify vegetables, and (b) improves the self‐reported preference for vegetables that have been cultured during these interventions. In addition, we expect an increased willingness to perform gardening, cooking, and outdoor activities.

## 2 MATERIALS AND METHODS

### 2.1 Characteristics of study groups

The study was conducted at three primary schools in the Municipality of Nijmegen (the Netherlands) and included 150 students of age 10–12 years. A control group of 65 students of the same year cohort from three other primary schools was used. All children of the same year cohort in all schools participated in either the intervention or the control group. All control group schools are located in the same area as the intervention group schools to avoid bias by socioeconomic background. An increase in sample size was thus not feasible, because it would require additional schools and thereby create more heterogeneity between the groups. However, a larger sample size was not expected to affect significance of results.

Both the intervention group (*n* = 150) and control group (*n* = 65) consisted of children with a mixed economic and cultural background. Nevertheless, their composition was similar regarding origin of children (63% vs. 64% autochthonous, respectively) and education level, employment, and income of their parents (low education level 11% vs. 9%, unemployment 8% vs. 5%, and low income 32% vs. 31%, respectively).

The schools were nonrandomly assigned to intervention groups. Randomization was not possible because only a limited number of schools were able to accommodate school gardening and research on effects of this intervention on students. Participation of schools was limited by several factors, such as (a) lessons in school gardening were not mandatory in educational curricula in the Netherlands, (b) lack of time or other preferences for obligatory components in curricula, (c) lack of budget, and (d) too large walking distance between garden and school.

### 2.2 Intervention approach

Over the periods of March–October 2015 and 2016, 76 and 74 students of three schools followed 17 lessons on gardening, respectively. In total, 150 different students participated in the program. All students followed the same course that consisted of one classroom lesson, 15 outdoor gardening lessons, and one harvesting and cooking lesson. Both the indoor lesson and outdoor sessions had a duration of 1 hr and were organized once a week. The classroom lesson focused on the life cycle of plants, identification of vegetables, and health aspects of eating vegetables. Students could also feel, smell, and taste the vegetables. The main activities during the outdoor gardening were: seeding, planting, weeding, and harvesting. During the outdoor sessions at the school garden, 11 different vegetables were planted and grown: lettuce, beetroot, zucchini, pumpkin, rucola, radish, potato, spinach, green bean, onion, and carrot. Students were encouraged to take the harvested vegetables home to cook and consume them. The final lesson was set‐up as a 3‐hr event, during which most of the vegetables were harvested and prepared for dinner. This lesson took place approximately 2 months after the last gardening session.

### 2.3 Quantitative variables

Both the intervention and control group responded to a questionnaire immediately before the first lesson and immediately after the last lesson of the intervention. The variables measured in the survey were as follows: (a) the capability to identify vegetables (12 questions), (b) the preference for those vegetables (14 questions), and (c) the opinion regarding vegetables, gardening, and outdoor activity (seven questions). In total, 106 of the 150 participants in the intervention responded to the survey both before and after the intervention. The control group consisted of 65 participants that filled out both surveys. The long‐term effect was studied by repeating the survey 1 year later among the groups that followed the project in 2015 (*n* = 52).

The survey consisted of 42 questions and took between 15 and 25 min to complete. For identifying vegetables, open questions were used. Children were asked to identify each of 12 unprocessed vegetables based on images. For vegetable preference and opinion regarding statements, Likert‐type questions were used. They had to rate their preference for 14 vegetables on a scale of 1 to 7, where 1 corresponds to “I do not like this vegetable at all” and 7 corresponds to “I like this vegetable a lot.” Instead, they could check the box with “do not know or have never tasted this vegetable.” Last, they had to judge seven statements about vegetables, gardening, and outdoor experience on a scale of 1 (“strongly disagree”) to 5 (“strongly agree”).

Cronbach's alpha for these three subsets of questions was, respectively, 0.83, 0.73, and 0.80, indicating that the internal reliability of the three categories of questions is acceptable to good. In the best case, multiple questions or statements would have been used to measure the same variable. However, the maximum length of the survey for the target group limited us to one item per variable.

The response per student for each vegetable was classified. Either the vegetable was known or unknown, depending on the capability to identify the vegetable. Second, either the vegetable was rated or not rated, depending on whether a preference rating was given or not. From this, frequency distributions were calculated using summed results per group, which were used to quantify students’ self‐knowledge of preferences. If students were unable to identify a specific vegetable, their preference for this vegetable was excluded from the results.

### 2.4 Statistical analyses

The nonparametric Wilcoxon signed‐rank test was used to analyze statistical significance of the effects of intervention on preference and attitude on statements. The sign test was used on the capability to identify vegetables. In both cases, the significance level (*α*) was 0.05. The Wilcoxon rank‐sum test (Mann–Whitney *U* test) (Fay & Proschan, 2010; Wilcoxon, 1945), a nonparametric test equivalent to the dependent *t* test, is appropriate when normality in the data cannot be assumed. This test is used to compare two sets of scores that come from the same group of participants and to compare the changes in the intervention group with the control group. Students were excluded from analysis when they could not participate in one or more of the surveys due to, for example, sickness, which resulted in a lower sample size for the analysis (*n* = 106 for short‐term survey, *n* = 56 for long‐term survey) than in the intervention (*n* = 150).

## 3 RESULTS

### 3.1 Capability to identify vegetables

**Figure 1:**
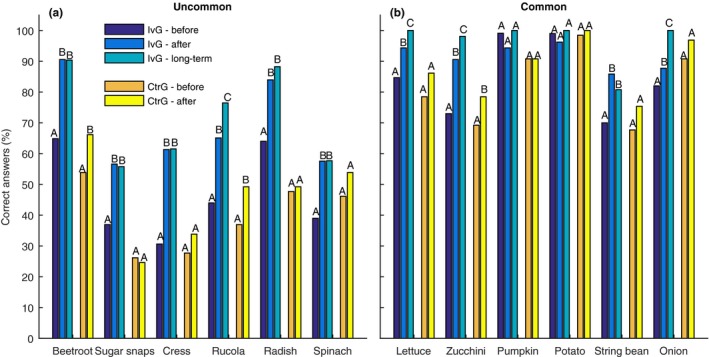
The capability of students to identify uncommon (a) and common known vegetables (b), indicated by a percentage of correct answers given in the survey. “IvG” indicates the treatment group, and “CtrG” indicates the control group. Different capital letters show significant changes between successive measurements (see also Table 1).

School gardening significantly increases the knowledge of primary schoolchildren on ten vegetables (Figure 1, Table 1). All these vegetables were significantly better identified immediately after the intervention (*n* = 106) as well as on the long term (*n* = 52). The capability to identify pumpkin and potato did not significantly increase, because these vegetables were already well‐known before the intervention (>98% correct). The increase in the number of students that accurately identified the vegetables was largest for the uncommon vegetables, which were the vegetables that less than 70% of the group identified correct before the intervention (Figure 1a). The capability to identify and name vegetables increased significantly even further 1 year after the intervention for rucola, lettuce, zucchini, and onion (Figure 1). For all other vegetables, the knowledge on the long term was approximately equal to the knowledge immediately after the intervention (Figure 1).

**Table 1:** The increase in the capability to identify vegetables and its significance. “All” includes the cumulative score on identifying all vegetables

	Intervention group				Control group	
	After *n*=106		Long term *n*=52		After *n*=65	
Vegetable	Change (%)	*p*	Change (%)	*p*	Change (%)	*p*
Lettuce	11	*<*0.01	18	*<*0.001	10	n.s.
Beetroot	40	*<*0.001	39	*<*0.001	23	<0.05
Zucchini	24	*<*0.001	34	*<*0.001	13	<0.05
Sugar snaps	53	*<*0.001	51	*<*0.05	−6	n.s.
Pumpkin	−5	n.s.	1	n.s.	0	n.s.
Cress	100	*<*0.001	101	*<*0.001	22	n.s.
Rucola	48	*<*0.001	74	*<*0.001	33	<0.05
Radish	31	*<*0.001	38	*<*0.001	3	n.s.
Potato	−3	n.s.	1	n.s.	2	n.s.
Spinach	48	*<*0.001	48	*<*0.05	17	n.s.
Green bean	23	*<*0.01	15	*<*0.05	11	n.s.
Onion	7	*<*0.05	22	*<*0.05	7	n.s.
**All**	**25**	***<*** **0.001**	**34**	***<*** **0.001**	**10**	**0.11**

*Note*

n.s.: not significant.

Scores of the treatment group on identifying vegetables increased significantly more than that of the control group (*p* < 0.001; Table 1). Nevertheless, an increase in knowledge was measured for the control group too (*n* = 65; Figure 1; Table 1), suggesting a possible learning effect for a few vegetables. For all vegetables, except sugar snaps, pumpkin, and potato, the control group filled in more correct answers during the second survey, which was held at the same time as when the treatment group ended the gardening intervention. However, only the increase for beetroot, zucchini, and rucola was significant and the combined results for all vegetables were not significant (*p* = 0.11; Table 1). When these scores are compared with the scores of the treatment group after the intervention, students in the control group only scored slightly higher on identifying potato. After correcting for the minor differences in the starting level of knowledge on vegetables, the increase in the number of correct answers and level of significance was higher for the intervention group (25%; *p* *<* 0.001) than for the control group (10%; *p* = 0.11) (Table 1).

### 3.2 Self‐reported preferences

The self‐reported preference for vegetables on average increased for the intervention group, being significant for beetroot, sugar snaps, cress, green bean, and carrot (Table 2). One year later, significant effects remained for carrot and preference for potato, onion, and tomato increased significantly compared with the measurement before the intervention (Table 2). The control group lacked an increase in self‐reported preference ratings (Table 2). Moreover, average preference ratings for the treatment group increased significantly more (*p* = 0.05) than for the control group (Supporting Information Table S2).

**Table 2:** Level of significance for the increase in self‐reported preference ratings

	Intervention group				Control group	
Vegetable	After		Long term		After	
	*n*_a_	*p*_a_	*n*_lt_	*p*_lt_	*n*_c_	*p*_c_
Lettuce	48	0.36	17	0.47	23	>0.50
Beetroot	27	***<*** **0.05**	9	0.16	7	0.45
Zucchini	37	0.71	18	0.14	19	0.50
Sugar snaps	19	***<*** **0.05**	9	0.09	6	0.11
Pumpkin	40	0.27	25	0.09	18	0.12
Cress	9	***<*** **0.05**	3	0.79	7	>0.50
Rucola	15	0.52	13	0.72	10	>0.50
Radish	33	0.10	11	0.76	8	>0.50
Potato	48	0.26	23	***<*** **0.05**	27	0.50
Spinach	14	1.00	4	0.64	10	0.43
Green bean	27	***<*** **0.05**	19	0.12	19	>0.50
Onion	37	0.07	26	***<*** **0.05**	29	>0.50
Tomato	46	0.21	22	***<*** **0.05**	26	>0.50
Carrot	49	***<*** **0.01**	30	***<*** **0.05**	34	0.31

*Notes*

Statistically significant increases are indicated in bold.

*n*: the number of students that changed their self‐reported preference compared to the survey before the intervention. *p*: statistical significance with the Wilcoxon rank‐sum test (Mann–Whitney *U* test).

The intervention increased both the students’ knowledge on vegetables and their ability to self‐report preference for the vegetables (Supporting Information Figure S1). The number of vegetables that was correctly identified and subsequently rated for preference increased in the intervention group. The long‐term measurement indicates that 78% of the responses concern vegetables that are both correctly identified and rated, which is higher than before the intervention (52%; Supporting Information Figure S1). In the control group, 30% of the self‐reported preferences are given for unknown vegetables, which is only 14% for the intervention group. The number of responses for vegetables that were known but not rated decreased from 11% to 6% for the intervention group. The decrease in the class of “unknown, not rated” is even larger (19% before to 5% on the long term).

### 3.3 Attitude toward vegetables, gardening, and cooking

The intervention had no significant effect on the attitude toward vegetables, gardening, and outdoor activity (Supporting Information Table S3). From written experiences about the intervention, it was noted that students found the gardening activities tougher than they expected, in particular weeding and hoeing. This also explains why the percentage of students that would like their own vegetable garden significantly decreased from 98% before the intervention to 67% on the long term (Supporting Information Table S4). This decline is probably due to an exceptionally high percentage in the initial survey, caused by excitement about the upcoming project. Most of the participants (87%) would like to participate in a school gardening project again.

## 4 DISCUSSION

### 4.1 Comparison with previous studies

This study shows that a school garden intervention significantly increases the capability of students to identify vegetables, which is in agreement with previous studies that tested the effect of an intervention on capability to identify vegetables (Davis, Martinez, Spruijt‐Metz, & Gatto, 2016; Koch, Waliczek, & Zajicek, 2006; Morris, Neustadter, & Zidenberg‐Cherr, 2001; Morris & Zidenberg‐Cherr, 2002; Parmer, Salisbury‐Glennon, Shannon, & Struempler, 2009; Ratcliffe et al., 2011). These effects remain on the long term, that is, 1 year after the intervention (Figure 1, Table 1). We also found an increase in scores for the control group too, which previously has only been reported by Davis et al. (2016) and thus suggests a learning effect over time (Edwards & Hartwell, 2002) or from performing a survey about vegetables. However, on average, for all vegetables the effects were not significant for the control group.

Multiple studies show that effects of gardening on preferences for vegetables are positive (Lineberger & Zajicek, 2000; Morgan et al., 2010; Morris & Zidenberg‐Cherr, 2002; Parmer et al., 2009; Triador, Farmer, Maximova, Willows, & Kootenay, 2015), but its significance is often lower than for the increase in knowledge. Moreover, Parmer et al. (2009) found that a school garden intervention only significantly increases taste ratings when students could taste the vegetable during the questionnaire, while their self‐reported preference of their fruit and vegetable preference remained stable. We found an increase in students’ capability to indicate their preferences for the intervention group: on the long term, this group rated more vegetables they knew but could not rate before the intervention and the number of ratings for vegetables they were unable to identify decreased as well (Supporting Information Figure S1).

Research on the positive effect of school gardening on preference for vegetables is primarily performed in the United States (Supporting Information Table S1), where the obesity problems are large. Our results as well as a study in Municipality of Amsterdam (the Netherlands) (Dijkstra, 2016; Nury et al., 2017) extend the observation of a positive effect of school gardening to continental northwestern European countries.

### 4.2 Limitations

The test for significance on the long term and for the control group is hampered by the number of participants for only one vegetable (potato), because in that case too few students changed in their capability to identify it. Cronbach's alpha was close to 0.70 for the questions about self‐reported preference, which indicates that the sample size of the long‐term measurement and control group should be increased to at least 100 to increase validity of these questions.

As the groups consisted of mixed cultural backgrounds, some students may have experienced difficulties with assessing their preference for vegetables based on their Dutch name, because they might only know the name in their native language. In addition, some vegetables can be experienced as unfavorable under raw or unprocessed conditions, such as onion, but may be preferred when prepared in a full meal. Last, most variables were measured with a single statement to limit the length of the questionnaire, for example, students’ attitudes regarding outdoor activity. This may limit the validity of the measured variable, because the type of outdoor activity may affect students’ attitude toward it. Therefore, we recommend for future research on the effect of school gardens: (a) to test for preferences based on imagery and if possible based on tastings during the survey (Parmer et al., 2009) to include taste ratings for unknown vegetables, (b) to assess the preference for full meals (Ahlström, Baird, & Jonsson, 1990; Noble, Corney, Eves, Kipps, & Lumbers, 2003) in which vegetables are used (e.g., pasta with vegetables) compared with meals without vegetables (e.g., pasta with ham and cheese) rather than individual vegetables, and (3) to include multiple statements that test the same variable in the questionnaire.

### 4.3 Implications

We conclude that school gardening programs increase students’ knowledge of vegetables and their self‐knowledge of preferences on the short term. On the long term, the increased involvement with vegetables at home and the gardening intervention itself increased the students’ exposure to vegetables, for which Birch et al. (1987) showed that it increases visual and taste preference for vegetables. An increase in preference may be one of the best indicators for future vegetable intake (Birch, 1979; Blanchette & Brug, 2005; Domel et al., 1996).

The lack of a positive effect on how students favor gardening and outdoor activity suggests that it may be necessary to revise the program of school garden interventions. Because students found some gardening tasks (e.g., weeding) harder than expected, it is recommended to enhance the gardening program with educational strategies to motivate students and keep their expectations realistic (Nury et al., 2017). We recommend to balance activities that are unfavorable, such as weeding and hoeing, with activities they consider more fun, such as seeding, harvesting, cooking, and tasting (Nury et al., 2017). It will be of interest to optimize the gardening intervention, such that it can also improve students’ attitudes toward nature, gardening, and green outdoor activities. Because the present‐day school gardening programs may increase future intake of vegetables, it is recommended to study their effect on other behavioral factors affecting human health, such as the daily indoor and outdoor activities of children.

## ETHICAL STATEMENT

The study was conducted in accordance with the ethical standards in the Helsinki Declaration of 1975, as revised in 2008. In the Netherlands, only the research projects that involve medical research on human subjects require registration with a Medical Ethics Review Committee (MERC) by law. Because this study is not of medical nature and did not involve sensitive topics nor exposure to any significant risks, Institutional Review Board Approval was not necessary. Consent was obtained from all participating schools, and parents were informed about the project and were given the possibility to withdraw their child from the project. Participation was voluntarily, and students were free to withdraw without consequences.

## CONFLICT OF INTEREST

The authors declare that they do not have any conflict of interests.
