# Supplementary material for: School gardening increases knowledge of primary school children on edible plants and preference for vegetables
Source: Food Sci Nutr. 2018 Sep 7;6(7):1960–7. doi: 10.1002/fsn3.758 (PMC6189627; doi:10.1002/fsn3.758)
Supplement: Supplementary file 1 [file FSN3-6-1960-s001.pdf]

Supporting Information for: *School gardening increases knowledge of primary school children on edible plants and preference for vegetables*

J.R.F.W. Leuven, A.H.M. Rutenfrans, A.G. Dolfing, R.S.E.W. Leuven

---

Contents

1. Figure S1: Percentages of students rating their preference for vegetables that they cannot identify and lack a preference for vegetables that they can identify.
2. Table S1: Overview of gardening interventions.
3. Table S2: Mean self-reported preference for vegetables.
4. Table S3: The degree to which students agree with given statements.
5. Table S4: Percentage of students that agreed with the statements.

*Corresponding author
Email address: j.r.f.w.leuven@uu.nl (J.R.F.W. Leuven)

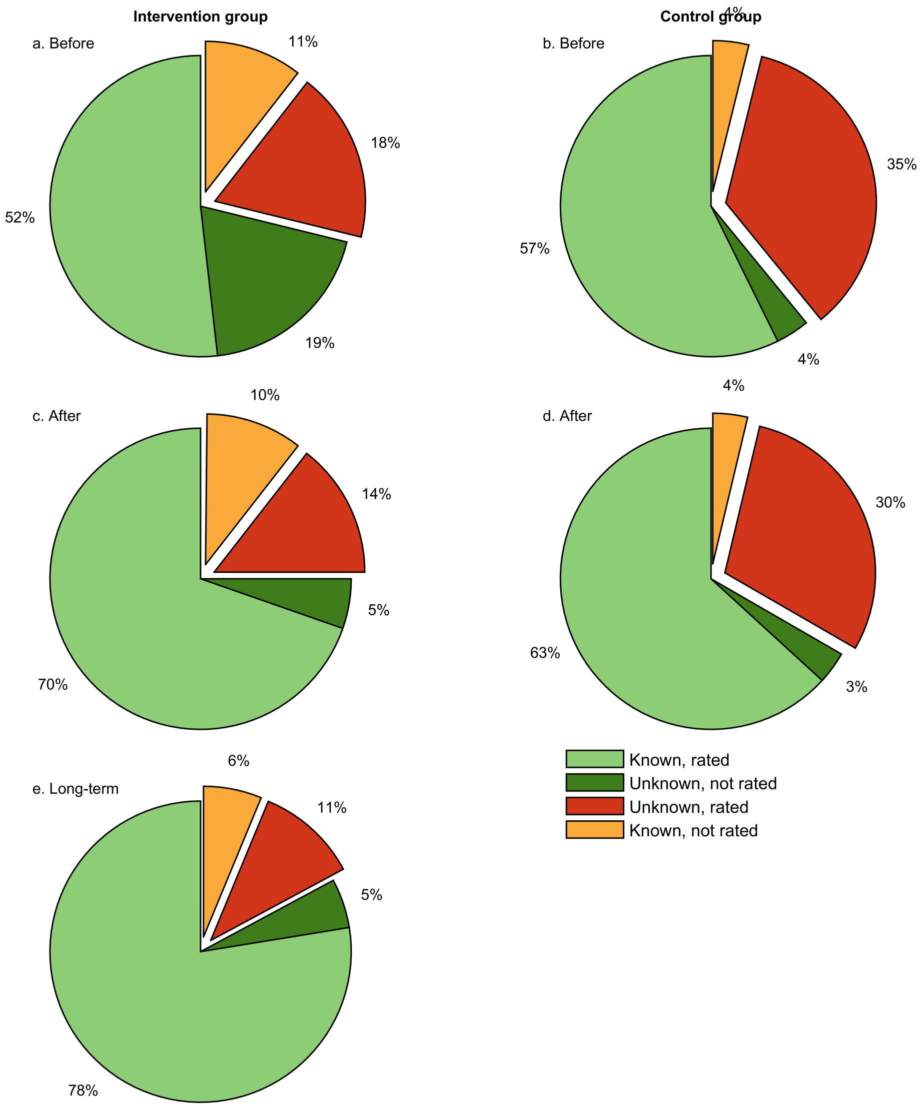

Figure 1: Percentages of students rating vegetables that they cannot identify and lack a self-reported preference rating for vegetables that they can identify, using summed measurements per group. The response per student for each vegetable was assigned one of the following categories: capable or incapable to identify the vegetable (known and unknown, respectively) and preference rating given or not given (rated and not rated, respectively).

Table 1: Overview of sample size, intervention duration and long-term monitoring of the effects, participant age and location of school gardening interventions.

Reference	n (interv)	t (interv)	long-term?	t (long-term)	Age (yr)	Continent	State or province	Comments
Davis et al. (2011)	34	12 weeks	No		9-11	North America	California (USA)	
Morris & Zidenberg-Cherr (2002)	81	17 weeks	Yes	6 months	9-10	North America	California (USA)	
Morgan et al. (2010)	35	10 weeks	Yes	4 months	11-12	Australia	New South Wales	
Wright & Rowell (2010)	234	3 weeks	No		5-11	North America	Wisconsin (USA)	No control
Ratcliffe et al. (2011)	170	17 weeks	No		11-13	North America	California (USA)	
Alexander et al. (1995)	30	n.a.	No		7-9	North America	Texas (USA)	Interviews only
Hermann et al. (2006)	43	n.a.	No		8-14	North America	Oklahoma (USA)	
Heim et al. (2009)	93	12 weeks	No		8-11	North America	Minesota (USA)	
Parmer et al. (2009)	39	28 weeks	No		7-8	North America	Alabama (USA)	
Davis et al. (2016)	167	12 weeks	No		8-11	North America	California (USA)	
Lineberger & Zajicek (2000)	111	n.a.	No		8-11	North America	Texas (USA)	
Triador et al. (2015)	116	30 weeks	No		6-14	North America	Alberta (Canada)	
Nury et al. (2017)	45	35 weeks	No		9-10	Europe	The Netherlands	Observation and interviews
This study	150	30 weeks	Yes	1 year	10-12	Europe	The Netherlands	

n (interv) n̄umber of participants in gardening intervention; t (interv) d̄uration of gardening intervention; long term? Ȳes if monitoring continues at least a month after intervention; t (long-term) d̄uration until long-term monitoring, measured from the end of intervention period; n.a. n̄ot available

Table 2: Average self-reported preference for vegetables given on a scale of 1 to 7, including standard deviations. The preference of vegetables on average increased significantly more (P=0.05, non-parametric Wilcoxon signed-ranks test) in the intervention group compared to the control group.

	Treatment group						Control group			
	Before (n=106)		After (n=106)		Long-term (n=52)		Before (n=65)		After (n=65)	
Vegetable	μ	σ	μ	σ	μ	σ	μ	σ	μ	σ
Lettuce	5.4	1.5	5.5	1.4	5.5	1.0	5.6	1.3	5.3	1.5
Beetroot	3.5	2.0	4.0	1.9	4.0	1.9	3.9	2.1	3.9	2.0
Zucchini	4.0	2.0	3.8	2.1	3.6	2.0	4.3	2.1	4.3	2.1
Sugar snaps	4.5	1.8	4.4	1.8	4.2	2.0	5.1	1.9	4.3	2.2
Pumpkin	3.7	2.1	4.0	2.1	4.1	1.8	3.6	2.4	3.7	2.1
Cress	5.2	1.6	5.2	1.9	5.0	1.5	4.3	2.1	4.1	1.9
Rucola	4.9	1.8	4.7	1.5	4.3	1.6	4.9	2.0	4.1	2.0
Radish	3.7	1.9	3.9	2.0	3.8	1.7	3.7	2.0	3.4	1.6
Potato	5.9	1.4	5.9	1.4	6.0	1.5	5.9	1.3	5.8	1.3
Spinach	5.6	1.8	5.2	1.9	5.6	2.0	4.8	2.3	4.9	2.3
Green bean	4.8	1.9	4.9	1.8	4.7	1.9	5.1	2.2	4.8	2.0
Union	4.1	2.0	4.2	2.0	4.3	1.9	4.1	2.0	3.8	2.0
Tomato	4.8	2.3	4.7	2.3	5.4	2.1	4.6	2.2	4.6	2.1
Carrot	5.4	1.9	5.8	1.6	5.9	1.4	5.4	1.9	5.6	1.4

Table 3: Mean (μ) and standard deviation (σ) for the degree to which students agree with the given statements on a scale of 1 (strongly disagree) to 5 (strongly agree).

	Treatment group								Control group				
	Before (n=106)		After (n=106)			Long-term (n=52)			Before (n=65)		After (n=65)		
Statement	μ	σ	μ	σ	P	μ	σ	P	μ	σ	μ	σ	P
I like vegetables	4.1	1.0	4.1	0.9	0.50	4.1	0.9	0.11	3.9	1.3	3.8	1.2	0.50
I like to garden	4.3	1.0	3.9	1.2	1.00	3.4	1.3	1.00	3.5	1.5	3.3	1.5	0.76
I like to go outside	4.6	0.7	4.6	0.7	0.50	4.4	0.8	0.80	4.0	1.5	3.8	1.5	0.76
I only eat vegetables I know	2.6	1.4	2.6	1.3	0.50	2.7	1.3	0.07	2.8	1.6	2.7	1.7	0.69

Table 4: Percentage of students that agreed with the statements. The asterisks (*) indicate a significant decrease in students that agreed with the statements, compared to before the intervention. Other changes and difference between control and intervention group where not significant.

	Treatment group			Control group	
Statement	Before (%)	After (%)	Long-term (%)	Before (%)	After (%)
I help with cooking at home	84	87	90	85	83
I would like to have my own garden at home	98	83*	67*	73	72
I help more often in the garden at home now		42	41		
I cooked with the vegetables at home		85			
I went to the schoolgarden outside class hours		35			
I would like to do the gardening project again		87			
